# Hyperbaric oxygen therapy suppresses hypoxia and reoxygenation injury to retinal pigment epithelial cells through activating peroxisome proliferator activator receptor‐alpha signalling

**DOI:** 10.1111/jcmm.17963

**Published:** 2023-09-20

**Authors:** Tzong‐Bor Sun, Kuo‐Feng Huang, Ko‐Chi Niu, Cheng‐Hsien Lin, Wen‐Pin Liu, Chao‐Hung Yeh, Shu‐Chun Kuo, Ching‐Ping Chang

**Affiliations:** ^1^ Department of Hyperbaric Oxygen Medicine Chi Mei Medical Center Tainan Taiwan; ^2^ Division of Plastic Surgery, Department of Surgery Chi Mei Medical Center Tainan Taiwan; ^3^ Department of Biotechnology and Food Technology Southern Taiwan University of Science and Technology Tainan Taiwan; ^4^ Department of Medicine Mackay Medical College New Taipei City Taiwan; ^5^ Department of Medical Research Chi Mei Medical Center Tainan Taiwan; ^6^ Division of Neurosurgery, Department of Surgery Chi Mei Medical Center Tainan Taiwan; ^7^ Department of Optometry Chung Hwa University of Medical Technology Tainan Taiwan; ^8^ Department of Ophthalmology Chi Mei Medical Center Tainan Taiwan

**Keywords:** age‐related macular degeneration, apoptosis, autophagy, hyperbaric oxygen, oxidative stress, PPARα, retinal pigment epithelial cell

## Abstract

Retinal ischemia followed by reperfusion (IR) is a common cause of many ocular disorders, such as age‐related macular degeneration (AMD), which leads to blindness in the elderly population, and proper therapies remain unavailable. Retinal pigment epithelial (RPE) cell death is a hallmark of AMD. Hyperbaric oxygen (HBO) therapy can improve IR tissue survival by inducing ischemic preconditioning responses. We conducted an in vitro study to examine the effects of HBO preconditioning on oxygen–glucose deprivation (OGD)‐induced IR‐injured RPE cells. RPE cells were treated with HBO (100% O_2_ at 3 atmospheres absolute for 90 min) once a day for three consecutive days before retinal IR onset. Compared with normal cells, the IR‐injured RPE cells had lower cell viability, lower peroxisome proliferator activator receptor‐alpha (PPAR‐α) expression, more severe oxidation status, higher blood‐retinal barrier disruption and more elevated apoptosis and autophagy rates. HBO preconditioning increased PPAR‐α expression, improved cell viability, decreased oxidative stress, blood‐retinal barrier disruption and cellular apoptosis and autophagy. A specific PPAR‐α antagonist, GW6471, antagonized all the protective effects of HBO preconditioning in IR‐injured RPE cells. Combining these observations, HBO therapy can reverse OGD‐induced RPE cell injury by activating PPAR‐α signalling.

## 1 INTRODUCTION

Age‐related macular degeneration (AMD) can cause blindness in elderly people, and proper therapies remain unavailable. In clinical settings, damaged retinal pigment epithelial (RPE) cells are one of the most common features for the diagnosis of AMD.^1^ Ageing RPE cells can be generated by incubating the human adult REP cell line‐19 (ARPE‐19 cells) with H_2_O_2_ to mimic oxidative stress or under oxygen–glucose deprivation (OGD) to mimic ischemia–reperfusion (IR) injury.^2^, ^3^ The RPE cells under IR injury displayed lower cell viability, higher apoptosis rates and more severe oxidative stress than normal cells. Hence, strategies for protecting RPE cells against IR injury may be particularly important in preventing the development or progression of AMD.

Peroxisome proliferator‐activated receptor‐alpha (PPAR‐α) exists in the RPE, outer nuclear layer, inner nuclear layer and ganglion cell layer and is essential for lipid metabolism and neuronal survival in the retina.^4^, ^5^ A previous study demonstrated that PPARα, but not PPARβ or PPARγ, is down‐regulated in diabetic retinopathy.^4^ Fenofibrate (a PPAR‐α agonist) alleviates apoptosis of capillary pericytes^6^, ^7^ or loss of retinal ganglion cells^8^ in the ischemic retina. This raises the possibility that PPAR‐α is significantly down‐regulated during IR injury to RPE cells.

It is well known that hyperbaric oxygen (HBO) treatment, the administration of 100% oxygen at atmosphere absolute (ATA) greater than 1 ATA, can enhance the antioxidant defences and slow the ageing process.^9^ HBO can improve IR tissue survival by inducing ischemic preconditioning responses.^10^ Previous results further show that pharmacological protection of RPE cells involves PPAR‐α.^11^ Therefore, our present study attempted to test the hypothesis that HBO protects against ischemic RPE cell injury via activation of PPAR‐α.

To deal with these questions, using ARPE‐19 cells (human RPE cell lines), we explored the role of PPAR‐α and the effect of HBO in the pathomechanism of IR‐related retinopathy, including reduced mitochondrial biogenesis, decreased cell viability, reduced PPAR‐α expression, increased oxidative stress, blood‐retinal barrier disruption, apoptosis and autophagy in vitro.

## 2 MATERIALS AND METHODS

### 2.1 Cell culturing

ARPE‐19 cells, a human RPE cell line, were purchased from the American Type Culture Collection (Cat no: CRL‐2302, ATCC) and cultured in Dulbecco's modified Eagle medium/F‐12 (DMEM/F‐12) supplemented with 10% fetal bovine serum (Thermo Fisher Scientific, Inc.), 2‐mM L‐glutamine, 100 μg/mL of streptomycin and 100 U/mL of penicillin. Cultures were maintained at 37°C humidified incubator with 5% CO_2_.

### 2.2 Oxygen–glucose deprivation and reoxygenation model

Based on a previous study, we used oxygen–glucose deprivation/reoxygenation (OGD/R) to study IR injury in ARPE‐19 cells.^12^ For the induction of OGD, cells were washed with PBS, switched to DMEM without glucose and serum and placed in 0.2% O_2_ hypoxia chamber controlled by a ProOxC system balanced with 5% CO_2_/95% N_2_ (Biospherix) for 48 h at 37°C. After the end of OGD, cells were resupplied with glucose‐containing medium and reoxygenated to a normoxic incubator under 5% CO_2_/95% air for 24 h. The control cells were maintained with growth medium and normoxia at 37°C for 72 h. Cells were treated with 2.5–10 μM GW6471 (PPAR‐α antagonist, Selleckchem) to investigate the underlying mechanism during ODG/R.

### 2.3 Hyperbaric oxygen treatment

For pre‐treatment of HBO, cells were placed in a custom‐made experimental chamber (MEDITATE CO., LTD.) with inside 100% O_2_ under 3 absolute atmospheres (ATA) for 90 min and intermittent air breaks for 5 min every 30 min of oxygen. HBO was administered once a day for three consecutive days. Control cells were incubated simultaneously under normobaric air (NBA: 21% O_2_ and 1 ATA).

### 2.4 Experimental groups

**FIGURE 1:**
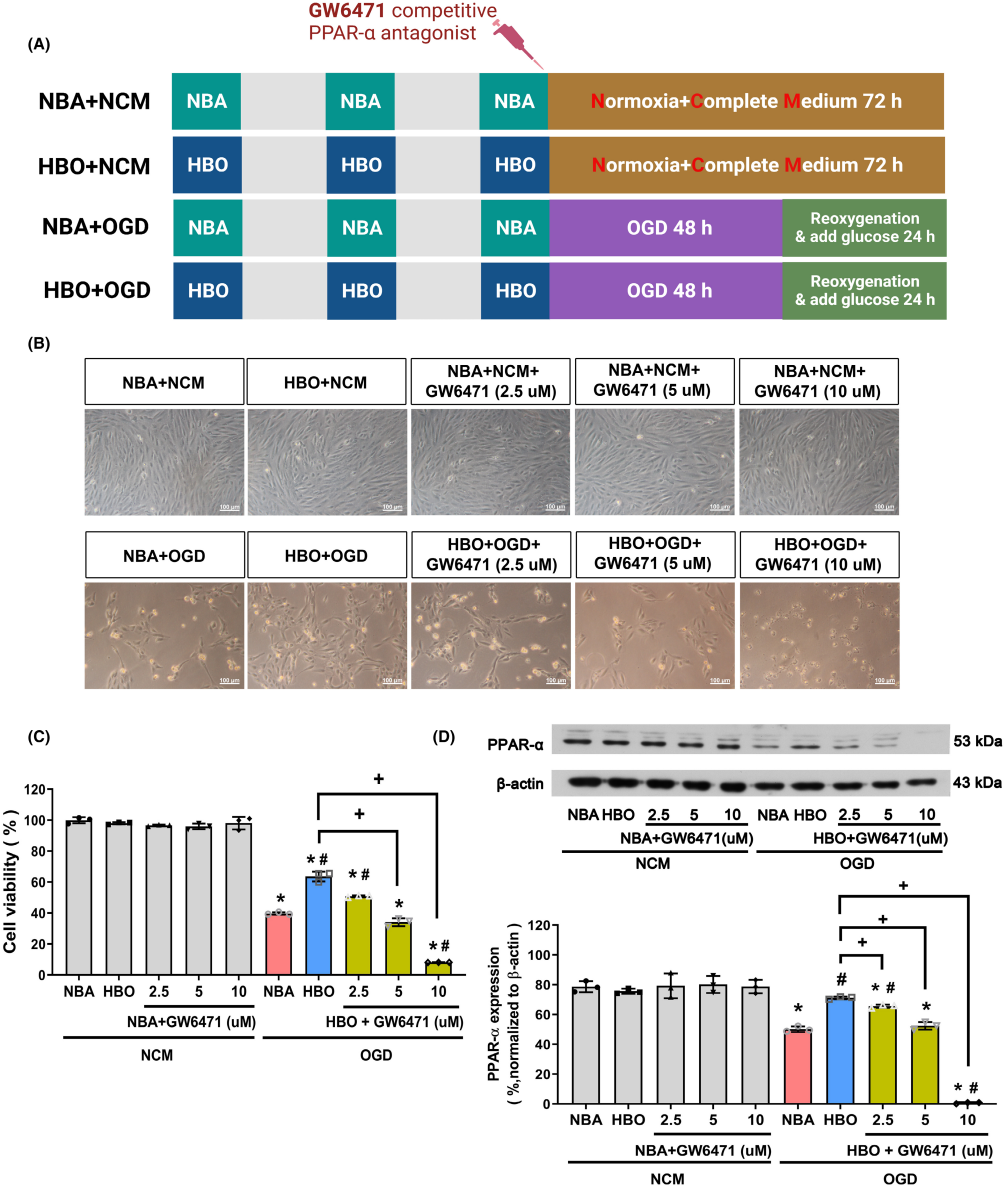
Activation of PPAR‐α is required to protect ARPE‐19 by HBOT. (A) Four different treatment groups and protocols were used for experiments. (B) Representative photographs of ARPE‐19 morphological changes under an inverted phase‐contrast microscope (×200) for different groups. (C) CCK‐8 analysis of relative cell viability was calculated from the optical density value at 472 nm against that of the control group. Data from three sets of independent experiments were quantified. (D) Western blot analysis of PPAR‐α in ARPE‐19 cells for different groups. β‐Actin served as the loading control. The figure depicts the densitometric analysis of the bands for each group. *p < 0.05, versus NBA + NCM; ^#^ *p* < 0.05, versus NBA + OGD; ^+^ *p* < 0.05, versus HBO + OGD. HBO, hyperbaric oxygen; NBA, normobaric air; NCM, normoxia with complete medium; OGD, oxygen–glucose deprivation.

We included six different treatment groups of ARPF‐19 cells in the present study. In group 1, the cells were pretreated with NBA (90 min) once daily for three consecutive days before the exposure to normoxia/complete medium (NCM; 72 h; NBA + NCM). The cells in the group 2 were pretreated with HBO (90 min/day for three days) and NCM (72 h; HBO + NCM). In group 3, the cells were pretreated with NBA (90 min/day for three days) and NCM plus GW6471 (72 h; NBA + NCM + GW6471). In group 4, the cells were pretreated with NBA (90 min/day for three days) and OGD (48 h), followed by reoxygenation (24 h; NBA + OGD). In group 5, the cells were pretreated with HBO (90 min/day for three days) and OGD (48 h), followed by reoxygenation (24 h; HBO + OGD). In group 6, the cells were pretreated with HBO (90 min/day for three days) and OGD plus GW6471 (48 h) and reoxygenation (24 h; HBO + OGD + GW6471). Figure 1A shows the experimental design. Antibodies and commercial kits used in the biochemical assay were summarized in Table 1.

**TABLE 1:** Antibodies and commercial kits used in the biochemical assay.

Antibody/kit	Company	Titre	Catalogue	Purpose
LC3B	Cell Signalling	1:5000	2775	WB
p62	Cell Signalling	1:5000	39749	WB
Caspase‐3	Cell Signalling	1:2000	9662	WB
Caspase‐9	Cell Signalling	1:2000	9508	WB
Phospho‐AKt (Ser473)	Cell Signalling	1:2000	4060	WB
Total Akt	Cell Signalling	1:2000	9272	WB
Phospho‐ERK	Cell Signalling	1:5000	4370	WB
Total ERK	Cell Signalling	1:5000	9102	WB
F‐Actin	Abcam	1:2000	Ab205	WB
ZO‐1	Abcam	1:5000	Ab216880	WB and IF
PPAR‐α	ABclonal	1:2000	A18252	WB
β‐Actin	Santa Cruz	1:5000	sc‐47778	WB
Rhodamine Phalloidin (F‐actin)	ThermoFisher Scientific	1:400	R415	IF
4′6‐diamidino‐2‐phenylindole (DAPI)	Thermo Fisher	1:25000	62247	IF
Alexa Fluor 488 (rabbit IgG)	Invitrogen	1:400	A21441	IF
Anti‐rabbit IgG, HRP‐linked antibody	Cell Signalling	1:2000	7074	WB
Anti‐mouse IgG, HRP‐linked antibody	Cell Signalling	1:2000	7076	WB
MitoScreen (JC‐1) kit	BD Biosciences	–	551302	Flow cytometry
ROS‐ID^®^ Total ROS/Superoxide detection kit	ENZO Life Sciences	–	ENZ‐51010	Flow cytometry
Superoxide Dismutase activity assay kit	Abcam	–	ab65354	Colorimetric analysis
Catalase activity assay kit	Abcam	–	ab83464	Colorimetric analysis
Glutathione peroxidase assay kit	Abcam	–	ab102530	Colorimetric analysis
Hydrogen peroxide assay kit	Abcam	–	ab102500	Colorimetric analysis
Lipid peroxidation (MDA) assay kit	Abcam	–	ab118970	Colorimetric analysis
Lipid hydroperoxide assay kit	Abcam	–	Ab133085	Colorimetric analysis

### 2.5 Cell viability assay

We used a Cell Counting Kit‐8 (CCK‐8) assay (Abcam) to determined cell viability. One hour after CCK8 solution incubation, the absorbance (at 450 nm on a MultiSkan GO microplate reader, Thermo Fisher Scientific) of treated cells//control cells ratio was determined.

### 2.6 Assessment of mitochondrial membrane potential

Briefly, 100 μL of a JC‐1 working solution was added to cells and incubated in the dark for 30 min at 37°C. Following incubation, cells were washed once and resuspended using 400‐μL staining buffer for analysis via the Novocyte flow cytometry (ACEA Biosciences). A total of 10,000 cells were measured using the FL‐1 channel (525 nm), and results were expressed as a percentage.

### 2.7 Measurement of transepithelial electrical resistance

To establish cell monolayers, 1 × 10^5^ of ARPE‐19 cells were grown on microporous filter inserts (0.4‐μm pore size, BD Biosciences), and the inserts were placed in six‐well culture plates. We used a Millicell‐Electrical Resistance System (ERS‐2; Merck Millipore) to measure transepithelial electrical resistance (TEER). The values were reported as Ω × cm^2^ and were calculated as (average resistance of well‐average resistance of the blank well) × 4.2 (the area of membrane).

### 2.8 Immunofluorescence stain

After treatment, the cells fixed with 4% paraformaldehyde were blocked non‐specific binding with 5% non‐fat milk in PBS for 1 h and then incubated with ZO‐1 antibody at 4°C overnight. The cells on slide were incubated with Alexa Fluor 488 secondary antibody, followed by staining rhodamine phalloidin to label F‐actin and DAPI. All slides were analysed with an Axio Imager A2 fluorescence microscope (Zeiss).

### 2.9 Western blot analysis

Total proteins were extracted by RIPA buffer containing protease and phosphatase inhibitors (Sigma‐Aldrich) and quantified by Bradford method (Bio‐Rad). Then, the protein extracts were boiled for 5 min in sample buffer, separated on SDS‐PAGE and transferred to PVDF membrane (Pall Corporation) using a wet‐transfer system (Bio‐Rad). The membranes were blocked in 5% non‐fat milk in PBS containing 0.05% Tween‐20 (PBS‐T) for 1 h at room temperature and then hybridized with total Akt, p‐Akt (Ser473), LC3B, p62, caspase‐3 and 9 (Cell Signalling Technology), ZO‐1, F‐actin, PPAR‐α and β‐actin antibodies for overnight at 4°C. After washing with PBS‐T, the membranes were incubated with appropriate secondary antibodies coupled to horseradish peroxidase (Cell Signalling Technology) for 1 h at room temperature, developed in the ECL Western detection reagents (PerkinElmer) and visualized on film. Protein bands were scanned and quantified using lmageMaster software (TotalLab, Amersham Biosciences).

### 2.10 Detection of superoxide anions

The intracellular oxidative status of cells was evaluated using a specific superoxide detection kit, according to the manufacturer's instructions. Briefly, the superoxide detection agent (100 μL) was added to the cell and incubated at 37°C in the dark for 30 min. Then, cells were resuspended with 200 μL of washing buffer and analysed by Novocyte flow cytometry with 488‐nm excitation and 620‐nm emission.

### 2.11 Analysis of lipid peroxidation

Flow cytometry determined lipid peroxidation using a sensitive cell‐based ratiometric lipid peroxidation assay. Add lipid peroxidation sensor to ARPE‐19, incubated for 30 min at 37°C with 5% CO_2_ cell incubator, and then washed three times with HHBS. The fluorescence of cells was measured by using flow cytometry (Ex/Em = 488 nm/530 nm).

### 2.12 Detection of lipid hydroperoxides

We used a commercially available lipid hydroperoxide assay kit to determine the formation of lipid hydroperoxides (LPO). Samples were centrifuged at 1500 *g* for 5 min at 0°C. The 500 μL of the bottom chloroform layer was collected and then mixed with the chloroform–methanol solvent. The sample mixtures were added with 50‐μL chromagen and incubated at room temperature for 5 min. The absorbance was obtained at 500 nm on a glass 96‐well plate.

### 2.13 Determination of malondialdehyde

The cell supernatants after homogenization were used for measuring cellular levels of malondialdehyde (MDA) using an MDA assay kit. Briefly, 200‐μg proteins were mixed with thiobarbituric acid (TBA) and heated for 1 h at 95°C. After cooling on ice, we measured the absorbance of the samples at 532 nm. The content of MDA was calculated by a standard concentration curve.

### 2.14 Detection of hydrogen peroxide

We used a hydrogen peroxide (H_2_O_2_) assay kit to measure the levels of H_2_O_2_ in ARPE‐19 cells. The OxiRed probe reacts with H_2_O_2_ to produce a coloured product. Following the experiment, cells were collected, lysed in an assay buffer and centrifuged. The supernatants were deproteinization by using 10 KD spin columns, and the resulting samples were collected. The 50 μL of the sample was mixed with 50 μL of the reaction mix and then incubated at room temperature for 10 min. The absorbance was read at 570 nm and calculated the H_2_O_2_ concentration according to a standard concentration curve.

### 2.15 Measurement of antioxidant enzyme activity (superoxide dismutase, catalase and glutathione peroxidase)

The superoxide dismutase (SOD) activity in lysed cells was measured by the inhibition of reduction in the water‐soluble tetrazolium, WST‐1 (2‐(4‐iodophenyl)‐3‐(4‐nitrophenyl)‐5‐(2,4‐disulfo‐phenyl)‐2H‐tetrazolium, monosodium salt), which produced a formazan dye upon reduction with a superoxide anion. According to the manufacturer's protocol, 20‐μL lysed supernatant was mixed with 200‐μL WST‐1 working solution and 20‐μL enzyme working solution. After 20 min of incubation at 37°C, the absorbance was read at 450 nm. The SOD activity (inhibition rate %) was calculated.

Catalase (CAT) activity was determined using a commercially available catalase activity assay kit. Cells were lysed in an assay buffer, and the supernatants were collected by centrifugation. The rate of decomposition of H_2_O_2_ was measured spectrophotometrically at 570 nm.

Glutathione peroxidase (GPx) activity in cells was spectrophotometrically detected using a GPx assay kit. The kit is based on reducing the oxidized glutathione coupled with NADPH oxidation. The reduction in NADPH was determined at 340 nm. Cell supernatants were collected after lysed and centrifugation. Samples and co‐substrate mixtures were added to wells. Finally, 10 μL of cumene hydroperoxide was added to all wells to initiate the reaction. The absorbance was read at 340 nm after 5 min.

### 2.16 Statistical analysis

Statistical analyses were performed with GraphPad Prism 8.0.2 software (GraphPad Software Inc.). The data were presented as the mean ± SD. The *p* values were calculated using one‐way variance analysis (anova) with Tukey's post hoc test. A *p* value of <0.05 was considered to indicate a statistically significant result.

## 3 RESULTS

### 3.1 ARPE‐19 under ischemia–reperfusion injury caused retinopathy

**FIGURE 2:**
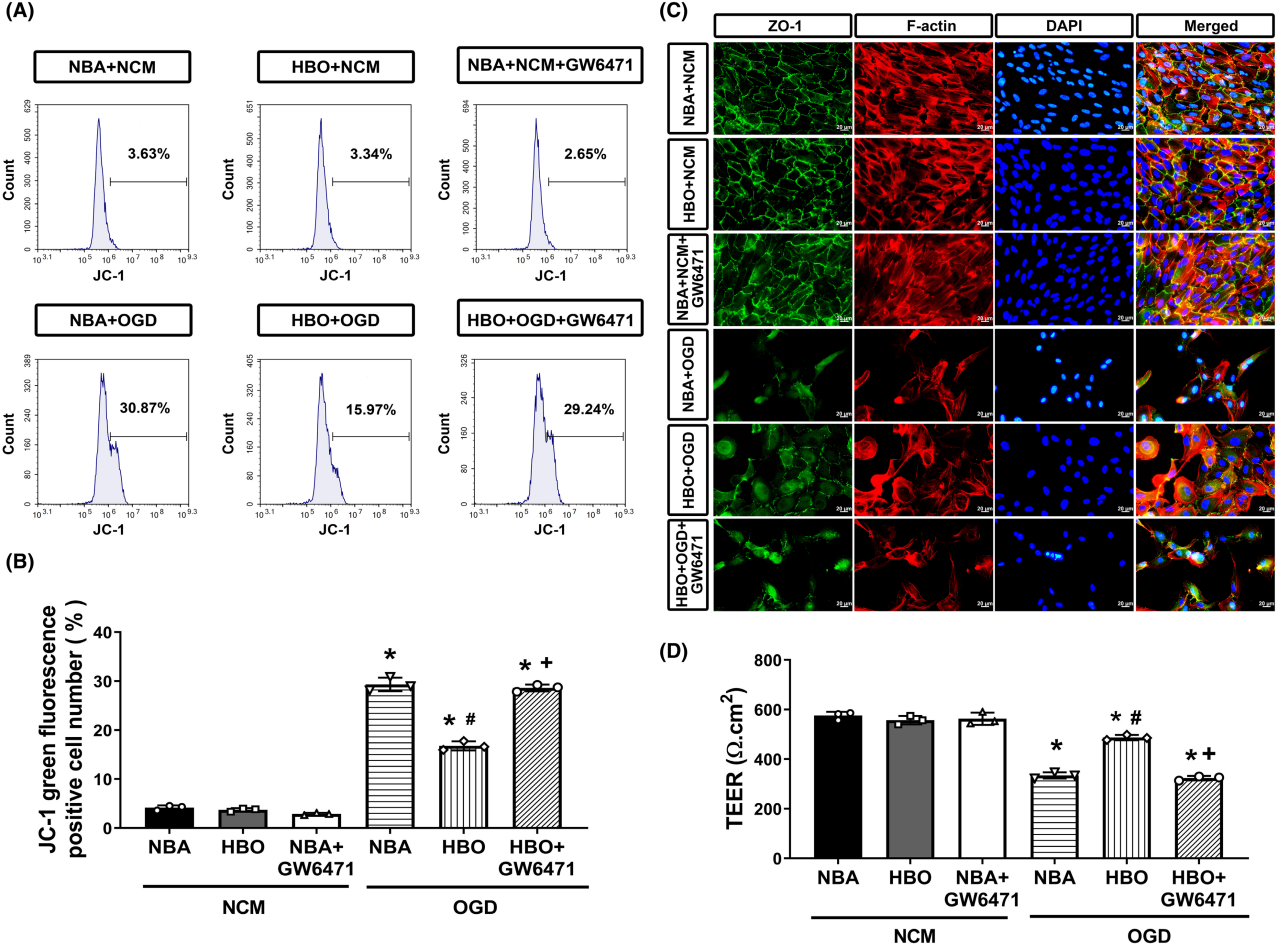
Protection by HBOT involves the reversal of the OGD‐induced decreased mitochondrial membrane potential (or increased JC‐1 green fluorescence by flow cytometry; A and B), (C) decreased ZO‐1 immunofluorescence and (D) decreased TEER in ARPE‐19 cells. Data from three sets of independent experiments were quantified. *p < 0.05, versus NBA + NCM; ^#^ *p* < 0.05, versus NBA + OGD; or ^+^ *p* < 0.05, versus HBO + OGD. HBO, hyperbaric oxygen; NBA, normobaric air; NCM, normoxia with complete medium; OGD, oxygen–glucose deprivation.

**FIGURE 3:**
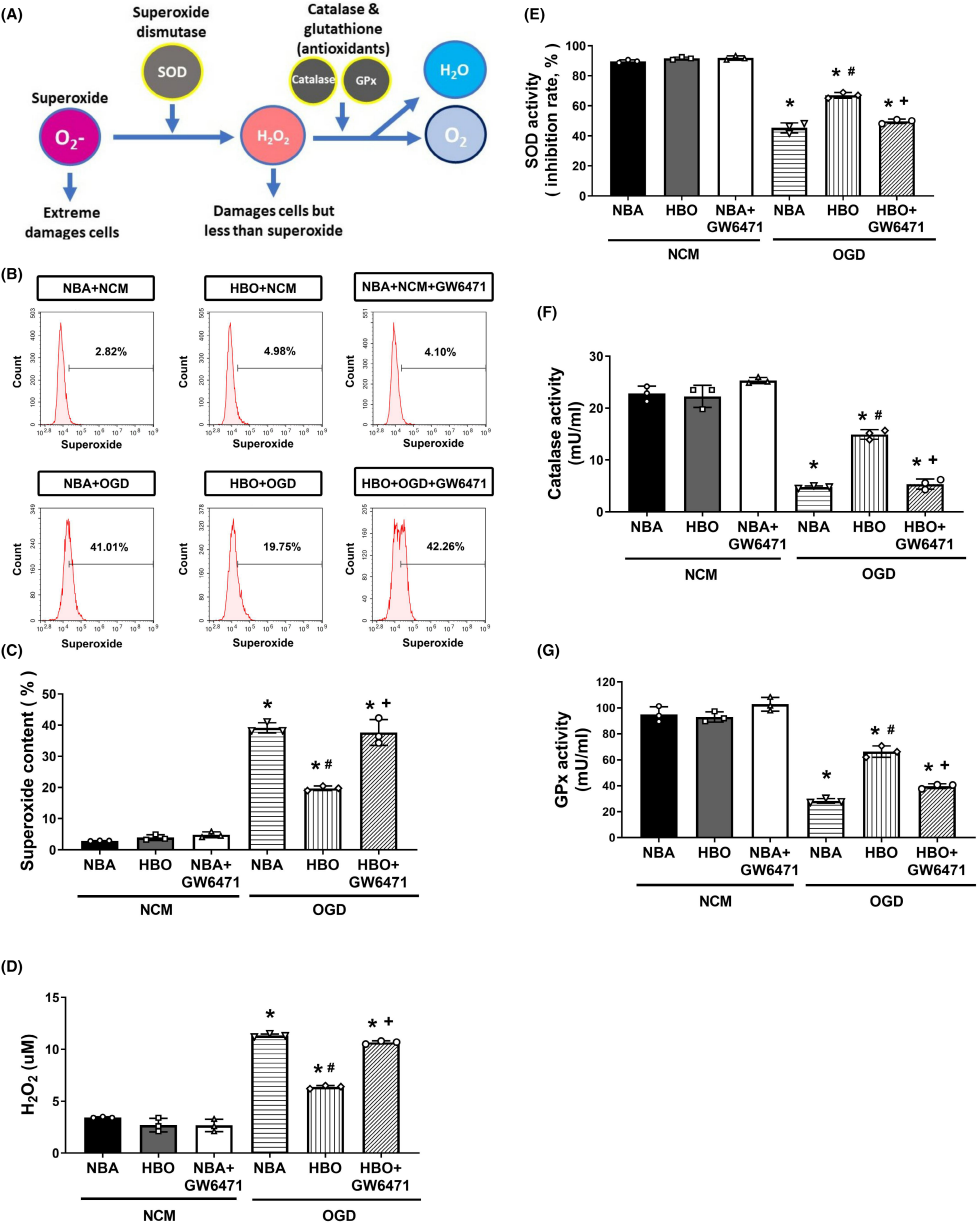
The HBO protection effect involves the reversal of the OGD‐induced oxidative stress‐related factor levels (A). The OGD‐induced (B and C) increased superoxide, (D) increased H_2_O_2_, decreased (E) SOD, (F) decreased catalase (G) and decreased GPx, in ARPE‐19 cells were attenuated by HBO. Data from three sets of independent experiments were quantified. *p < 0.05, versus NBA + NCM; ^#^ *p* < 0.05, versus NBA + OGD; and ^+^ *p* < 0.05, versus HBO + OGD. HBO, hyperbaric oxygen; NBA, normobaric air; NCM, normoxia with complete medium; OGD, oxygen–glucose deprivation.

**FIGURE 4:**
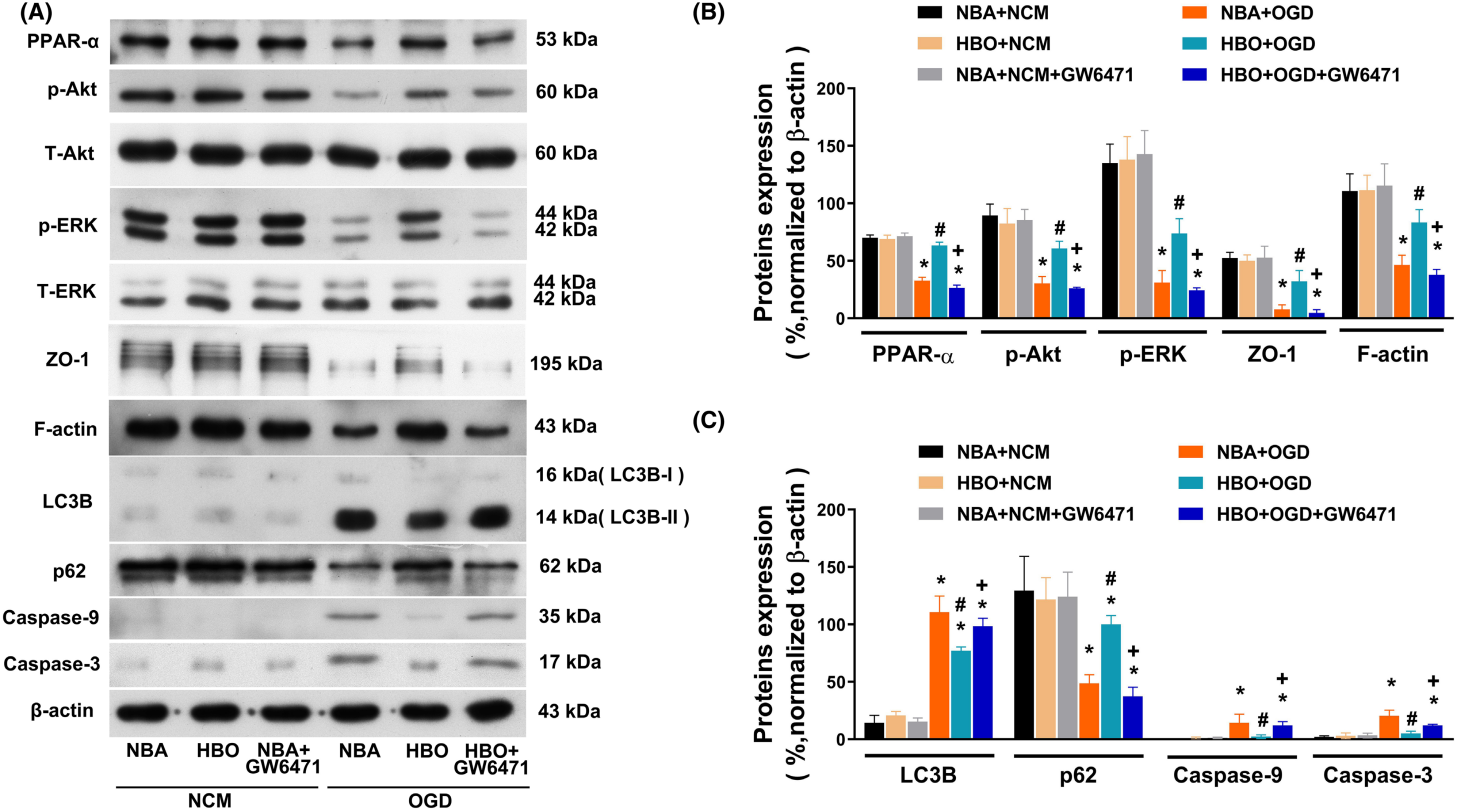
The HBO protection effect involves the reversal of the OGD‐induced decreased PPAR‐α, decreased p‐AKT, decreased p‐ERK, decreased ZO‐1, decreased F‐actin, increased, LC3B, decreased P62, increased caspase‐9 and increased caspase‐3 expression in ARPE‐19 cells. (A) Representative immunoblots are shown. β‐Actin was used as a loading control. (B and C) Measurements were made in triplicate, and each bar represents the mean ± SD *p < 0.05, versus NBA + OGD; ^#^ *p* < 0.05, versus NBA + OGD; ^+^ *p* < 0.05, versus HBO + OGD. HBO, hyperbaric oxygen; NBA, normobaric air; NCM, normoxia with complete medium; OGD, oxygen–glucose deprivation.

The dependence of ARPE‐19 cell viability on substrate support was evaluated through measurements of the effect of oxygen/glucose deprivation (OGD)‐induced ischemia/reperfusion (IR) injury on cell viability. IR injury significantly reduced cell viability in a time‐dependent manner. After 12 h of OGD, cell viability decreased to 90% and 70% as well as 50% after OGD for 36 h and 48 h, respectively (data not shown). When compared with normobaric air (21% O_2_ at 1.0 ATA; NBA) and normoxia/complete medium‐treated cell group (NBA + NCM), the group cells treated NBA and OGD (NBA + OGD) after OGD for 48 h had lower cell viability (Figure 1), lower PPAR‐α expression (Figure 1), lower mitochondrial activity (Figure 2), higher RPE permeability (Figure 2), higher superoxide (^·^O_2_^−^) levels, higher hydrogen peroxide (H_2_O_2_) levels, lower superoxide dismutase levels, lower catalase levels, lower glutathione levels (Figure 3), lower levels of p‐AKT levels, p‐ERK levels and p62 levels and higher levels of LC3B levels, caspase‐9 levels and caspase‐3 levels (Figure 4). Our results show that retinal ischemia followed by reperfusion causes retinopathy.

### 3.2 Activation of PPAR‐α by HBO before OGD protects ARPE‐19 cells from OGD‐induced IR injury

The experimental procedures in each group are shown in Figure 1A. The cell morphology observations (Figure 1B) and Cell Counting kit‐8 (CCK‐8) assay revealed that the number of cells in the NBA + OGD condition declined, and their morphology was altered compared to that in the NBA + NCM counterpart (Figure 1B,C). HBO preconditioning significantly reversed declines in the NBA + OGD group. On the other hand, 10‐μM GW6471 supplementation reversed the beneficial effects of HBO on cell viability (Figure 1C). Compared to the NBA + NCM group cells, both the cell viability and PPAR‐α expression values in the group cells treated with HBO and NCM (HBO + NCM) or HBO + NCM + GW6471 group cells were insignificantly different (*p* > 0.05, Figure 1C,D). However, compared to the NBA + NCM group, the values of both the cell viability and PPAR‐α expression in NBA + OGD group cells were significantly lower (*p* < 0.05, Figure 1C,D). The HBO + OGD group cells had significantly higher values of both the cell viability and the PPAR‐α expression than did the NBA + OGD group cells (*p* < 0.05, Figure 1C,D). Compared to the HBO + OGD group cells, HBO + OGD + GW6471 group cells had significantly lower values of both the cell viability and the PPAR‐α expression (*p* < 0.05, Figure 1C,D). Therefore, HBO preconditioning provides a protective effect against OGD‐induced decline in both PPAR‐α expression levels and ARPE‐19 cell viability.

### 3.3 HBO‐suppressed OGD‐mediated declines in mitochondrial activity and barrier function of ARPE‐19 cells

The mitochondrial membrane potential (MMP) difference and TEER values of the NBA + NCM group were not different than those in either the HBO + NCM or the NBA + NCM + GW6471 group (*p* > 0.05, Figure 2). However, compared to the NBA + NCM group cells, the NBA + OGD group cells had significantly higher values of JC‐1 green fluorescence (or decreased MMP; *p* < 0.05, Figure 2A,B) but lower values of TEER (*p* < 0.05; Figure 2D). Additionally, compared to the NBA + OGD group, the HBO + OGD group cells had significantly higher TEER values but significantly lower JC‐1 green fluorescence values (*p* < 0.05). However, compared to the HBO + OGD group cells, the HBO + OGD+ GW6471 (10 μM) group cells had significantly higher values of JC‐1 green fluorescence but lower values of TEER (*p* < 0.05, Figure 2B,D).

Both immunofluorescence stainings and western blotting assay revealed that compared to the NBA + NCM group cells, the NBA + OGD cells had significantly lower values of ZO‐1 protein (Figure 2C). Compared to the NBA + OGD cells, the cells treated with HBO + OGD had significantly higher values of ZO‐1 proteins (Figure 2C). The beneficial effects of HBO in elevating the values of ZO‐1 proteins were significantly reversed by GW6471 (10 μM) in the HBO + OGD+ GW6471 group cells (Figure 2C). Our data demonstrate that HBO reverses the OGD‐induced altered mitochondrial activities and RPE tight junction disruption.

### 3.4 HBO reverses rises in the superoxide ^·^O_2_ and increased H_2_O_2_ levels as well as declines in superoxide dismutase, catalase and glutathione content

It is known that exposure to oxidative stress can compromise cell function. Accordingly, we hypothesized that OGD‐induced cellular damage through generating rises in ^·^O_2_^−^ and H_2_O_2_ levels and decreased SOD, catalase and GPx content (Figure 3A). There was an insignificant difference between the NBA + NCM, HBO + NCM and NBA + NCM + GW6471 in the values of ^·^O_2_^−^, H_2_O_2_, SOD, catalase and GPx (Figure 3). However, compared to the NBA + NCM group, the NBA + OGD group had significantly lower values of SOD, catalase and GPx (Figure 3E–G, *p* < 0.05) but significantly higher values of both ^·^O_2_^−^ and H_2_O_2_ (*p* < 0.05, Figure 3C,D). However, the HBO + OGD group had significantly (*p* < 0.05) higher values of SOD, catalase and GPx but significantly (*p* < 0.05) lower values of both ^·^O_2_^−^ and H_2_O_2_ (Figure 3). Again, compared to the HBO + OGD group cells, the cells treated with HBO+ OGD + GW6471 group cells had significantly (*p* < 0.05) lower values of SOD, catalase and GPx but significantly (*p* < 0.05) higher values of both ^·^O_2_^−^ and H_2_O_2_ (Figure 3). Therefore, HBO reverses the OGD‐induced oxidative stress in ARPE‐19 cells.

### 3.5 HBO‐reversed OGD‐mediated declines in PPAR‐α, p‐AKT, p‐ERK, ZO‐1, F‐actin and P62 and rises in LC3B, caspase‐9 and caspase‐3 expression in ARPE‐19 cells

The western blotting assays (Figure 4A) showed that, compared to the NBA + NCM group cells, the NBA + OGD group cells had significantly lower values of PPAR‐α, p‐AKT, p‐ERK, ZO‐1, F‐actin and P62 but higher values of LC3B, caspase‐9 and caspase‐3 (Figure 4, *p* < 0.05). However, compared to NBA + OGD group cells, the HBO + OGD group cells had significantly higher values of PPAR‐α, p‐AKT, p‐ERK, ZO‐1, F‐actin and P62 but significantly lower values of LC3B, caspase‐9 and caspase‐3 (Figure 4). Again, compared to the HBO + OGD group cells, the HBO + OGD + GW6471 group cells had significantly lower values of PPAR‐α, p‐AKT, p‐ERK, ZO‐1, F‐actin and P62 but significantly higher values of LC3B, caspase‐9 and caspase‐3 (Figure 4, *p* < 0.05). For original images of the blots, please see Figures S1 and S2. Therefore, HBO reverses the OGD‐induced declines in PPAR‐α, p‐ERK, F‐actin and P62 and increases apoptosis in ARPE‐19 cells.

### 3.6 HBO failed to reverse OGD‐induced increases in lipid peroxidation in ARPE‐19 cells

**FIGURE 5:**
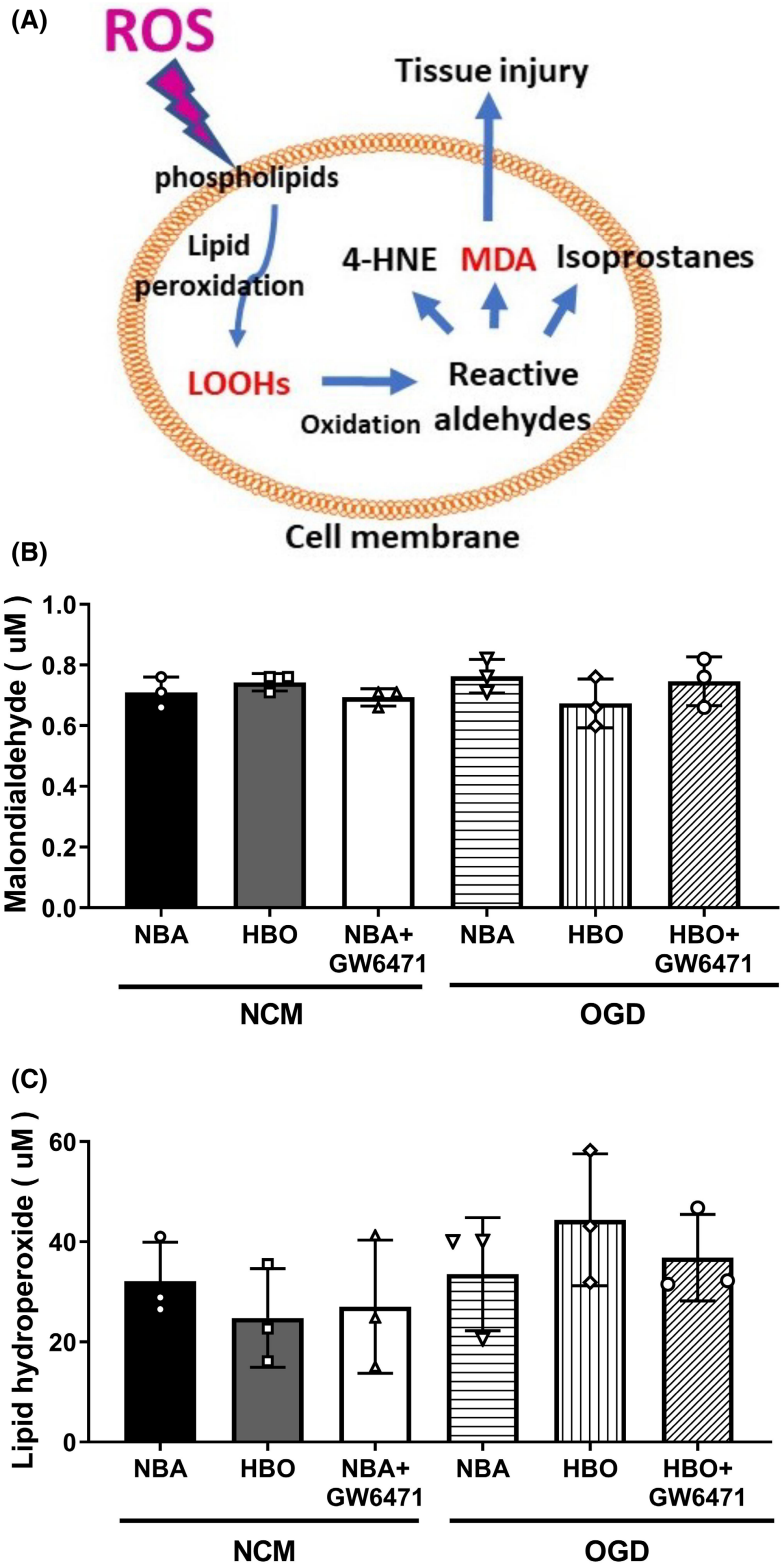
The HBOT protection effect does not involve lipid peroxidation. (A) The increased reactive oxygen species (ROS) generation might change the cell membrane phospholipids into LOOHs via undergoing lipid peroxidation (LPO) in OGD‐treated ARPE‐19 cells. ROS can induce the production of lipid peroxides (LOOH), 4‐Hydroxynonenal (4‐HNE), isoprostanes and malondialdehyde (MDA), which are compounds produced during lipid damage. (B) Malondialdehyde (MDA) assay comparing the lipid peroxidation in ARPE‐19 cells with different treatments. (C) Lipid hydroperoxide assay comparing the formation of highly reactive and unstable lipid hydroperoxide (LPO) of both saturated and unsaturated lipids in ARPE‐19 cells with different preparations. Data from three sets of independent experiments were quantified. HBO, hyperbaric oxygen; NBA, normobaric air; NCM, normoxia with complete medium; OGD, oxygen–glucose deprivation.

As shown in Section 3.4, OGD increased ^·^O_2_^−^ and H_2_O_2_ generation, suggesting the phospholipids might be undergoing lipid peroxidation in OGD‐treated ARPE‐19 cells (Figure 5A). Therefore, we examined lipid peroxidation in response to OGD, HBO and/or GW6471. Both the MDA assay and LPO assay revealed that the values of both MDA and LPO in the NBA + OGD group cells were insignificantly different from those of the NBA + NCM group cells (Figure 5B,C). Thus, the OGD‐induced increases in liquid peroxidation in ARPE‐19 cells were insignificantly affected by HBO. Therefore, inhibiting PPAR‐α inhibited the dissipation of oxidative stress since catalase and GPx expression levels were lower. These declines are consistent with ^·^O_2_^−^ and H_2_O_2_ levels being higher than in the absence of GW6471.

## 4 DISCUSSION

**FIGURE 6:**
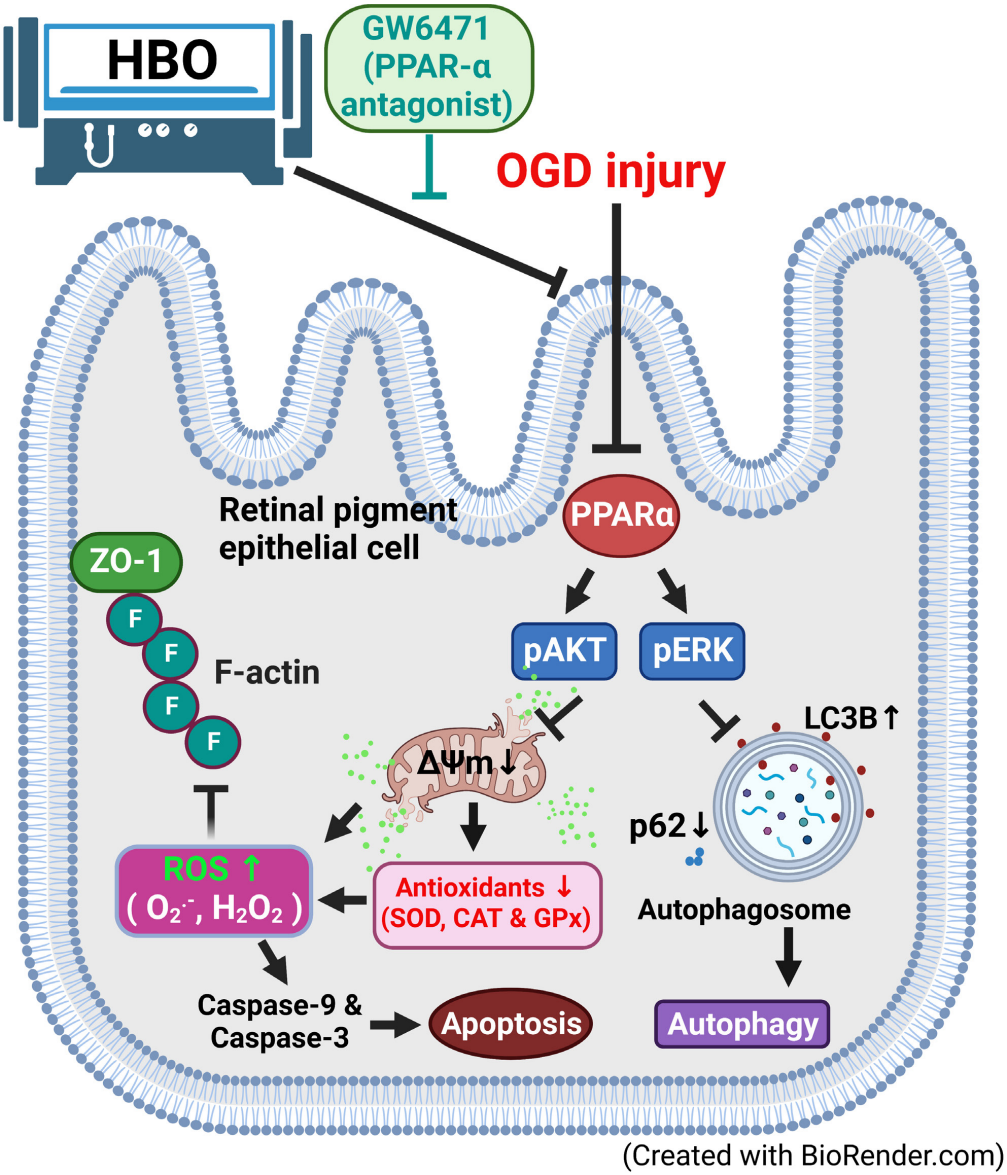
Schematic view of the signalling pathways elicited in ARPE‐19 cells under oxygen–glucose deprivation (OGD) conditions. OGD inhibited the PPAR‐α mediated p‐AKT (and resulted in decreased mitochondria membrane potential and decreased antioxidants), increased ROS generation and broke tight junction proteins (e.g. F‐actin and ZO‐1) and induced caspase‐9/‐3‐mediated apoptosis. PPAR‐α downregulation under OGD also decreased p‐ERK expression and LC3B activation, resulting in autophagy. Hyperbaric oxygen (HBO) preconditioning could attenuate the OGD‐induced apoptosis and autophagy signalling pathway by normalizing the PPAR‐α expression. A specific PPAR‐α antagonist, GW6471, can antagonize all the protection effects exerted by HBO in OGD‐injured retinal pigment epithelial cells.

First, when compared with normal (NBA + NCM) cells, we observed that changes induced by IR injury in ARPE‐19 cells were similar to those caused by H_2_O_2_.^13^, ^14^ Both of these stresses led to decreased cell viability, higher apoptotic rate and more severe oxidative stress. In addition, they display lower PPAR‐α expression, higher blood‐retinal barrier disruption (e.g. decreased tight junction protein expression), and higher autophagy (e.g. increased LC3B). Second, we show that HBO improves cell viability and reduces rises in both oxidative stress levels and blood‐retinal barrier permeability in ARPE‐19 cells under IR injury (as depicted in Figure 6). To verify whether the protection by HBO is dependent on a change in PPAR‐α function, we used a specific PPAR‐α antagonist, GW6471, to antagonize PPAR‐α and found that HBO exerts its protection in IR‐induced retinopathy via preserving PPAR‐α expression in situ. Treatment of the normal ARPE‐19 cells with either HBO or GW6471 alone did not change ^·^O_2_^−^ radicals and H_2_O_2_, SOD, catalase or GPx. However, OGD instead changed these values. This is an important point showing that OGD is perhaps a more effective inhibitor of PPAR‐α activation than GW6471 (please see Figure 1). Indeed, as indicated in Figure 1C,D, compared to NBA + NCM group cells, the NBA + NCM + GW6471 group cells have an insignificant effect on both the cell viability and PPAR‐α expression. Since GW6471 has a smaller inhibitory effect on control cells than do the OGD, this difference could suggest that GW6471 has other non‐specific effects besides inhibitory PPAR‐α activation.

Our findings are supported by several previous studies. Retinal ischemia–reperfusion down‐regulated PPAR‐α expression in vitro and in vivo.^4^, ^15^ Fenofibric acid (a PPAR‐α agonist) treatment promoted survival of ischemic retinal cells via enhancing the expression of endogenous PPAR‐α and promoting survival of retinal ganglion cells and mitigating thinning of the ganglion cell complex.^8^ Overexpression of PPAR‐α ameliorated both retinal inflammation and retinal vascular leakage via alleviating retinal neovascularization in diabetic retinopathy.^4^, ^16^ Fenofibrate therapy also alleviated retinopathy by reducing retinal inflammation and oxidative stress in both the ischemic retina^7^ and age‐related macular degeneration.^17^

The mitochondrion is implicated in the production of reactive oxygen species (ROS). The mitochondrial respiratory chain serves as the primary site of ROS production in the formation of ^·^O_2_^−^, H_2_O_2_ and the hydroxyl radical. Cytosolic superoxide is converted into H_2_O_2_ by superoxide dismutase. H_2_O_2_ can be detoxified to H_2_O by the scavenging enzymes GPx and catalase. In addition, H_2_O_2_ can react with metal cations to generate hydroxyl radicals and result in oxidative damage to lipids, proteins and DNA. As shown in the present results, OGD damaged ARPE‐19 cells via inducing mitochondrial distress (evidenced by decreased TEER and increased JC‐1 green fluorescence) and oxidative stress imbalance (evidenced by decreased SOD, decreased catalase and decreased GPx and increased superoxide and H_2_O). Accordingly, HBO might protect ARPE‐19 cells from I/R injury via alleviating mitochondrial distress and oxidative stress.

Previous studies have shown that oxidative stress‐induced RPE cell apoptosis is involved in the pathogenesis of age‐related macular degeneration.^18^, ^19^ In RPE cells, oxidative stress triggered degeneration via multiple cell death pathways, including P53‐regulated apoptosis,^20^ necroptosis^21^ and pyroptosis.^22^ In our study, OGD‐induced disruption of cell function is at least caused by both apoptosis (evidenced by increased expression of both caspase‐3 and caspase‐9) and autophagy (evidenced by increased expression of LC3B).

Previous studies showed that a PPAR‐γ agonist 15‐d‐prostaglandin J2 decreased neuronal autophagy by reducing the expression of proteins LC3B, Beclin‐1, cathepsin and LAMP1 in cerebral IR injury.^23^ We further demonstrated that HBO ameliorated both neuronal apoptosis and autophagy in IR‐injured ARPE‐19 cells caused by OGD‐induced IR condition via preserving physiological levels of PPAR‐α. Therefore, intrinsic PPAR pathways may be necessary for limiting injury, as PPAR deficiency induced by OGD process was associated with cell apoptosis, pyroptosis and autophagy. Protection of ARPE‐19 cells by HBO therapy involves reversal declines in PPAR‐α induced by OGD.

In our present model, ARPE‐19 cells under IR conditions display blood‐retinal barrier disruption via reducing tight junction proteins (such as ZO‐1), transepithelial electric resistance and mitochondrial membrane potential in ARPE‐19 cells. The IR might induce retinopathy via inducing oxidative stress and mitochondrial distress. Our data further show that HBO can attenuate the blood‐retinal barrier disruption by preserving the normal levels of ZO‐1 tight junction protein, TEER and MMP in ischemic ARPE‐19 cells.

In our HBO protocols, ischemic ARPE‐19 cells were exposed to not only breathing 100% oxygen under a high atmosphere but also intermittent fluctuations in oxygen. These fluctuations occur between the daily HBO as oxygen levels return from 100% to 21% at the end of the daily treatment. During each HBO, oxygen levels change from the physiological 21% oxygen to 100% oxygen and back to physiological oxygen levels of 21% several times. Key to the beneficial effects of HBO is the hyperoxic‐hypoxic paradox (HHP).^24^, ^25^, ^26^, ^27^, ^28^ Our present results further provide new evidence showing that intermittent HBO can improve ARPE‐19 cell survival under IR conditions via PPAR‐α activation.

Sirt1 is associated with regulating apoptosis, inflammation and senescence in ageing‐related diseases.^29^, ^30^, ^31^, ^32^, ^33^ A decrease in the Sirt1 results in the stabilization of HIF‐1α, while an increase in Sirt1 results in the activation of HIF‐1α,^34^ Sirt1 mediates such control through transducing interactions between oxygen and redox‐responsive signalling pathways.^35^, ^36^ Under normal conditions, higher ROS/ROS scavengers cause degradation of HIF‐1α,^28^ while in hypoxia, lower ROS/ROS scavengers cause accumulation of HIF‐1α as well as active HIF‐1α promotor. A single hyperoxic exposure and repeated hyperoxic exposure cause higher and lower ROS/ROS scavengers, respectively.^15^, ^37^, ^38^, ^39^, ^40^ Upon return to normoxia following repeated hyperoxic exposures, the ratio of ROS/scavenging capacity is low. Meaning intermittent hyperoxia decreases the capacity of ROS/ROS scavengers. Previous results showed that HBO causes ischemic tolerance via upregulation of HIF‐1α.^41^, ^42^ Our present data further show that intermittent HBO attenuates ischemia/reperfusion injury via activation of PPAR‐α as well as reduction in mitochondrial distress, blood‐retinal barrier disruption, apoptosis and autophagy in ARPE‐19 cells. The beneficial effects exerted by HBO all can be reversed by GW6471 therapy.

Evidence has accumulated to support the involvement of lipid peroxidation in diabetic retinopathy, age‐related macular degeneration, cataract formation, glaucoma and other eye diseases.^43^ Ferroptosis, a non‐apoptosis cell death, displays increased lipid peroxidation.^44^ However, our present study shows that IR‐induced cell death is associated with apoptosis and autophagy without lipid peroxidation. Our current results show that HBO therapy can reverse OGD‐induced RPE cell injury by activating PPAR‐α signalling. Additional studies for better understanding the beneficial effects of HBO and minimizing side effects are also necessary for optional clinical application in AMD.

## AUTHOR CONTRIBUTIONS

**Tzong‐Bor Sun:** Investigation (lead); methodology (lead); project administration (lead); writing – original draft (lead). **Kuo‐Feng Huang:** Formal analysis (equal); methodology (equal); supervision (equal). **Ko‐Chi Niu:** Data curation (equal); methodology (supporting). **Cheng‐Hsien Lin:** Data curation (equal); investigation (supporting). **Wen‐Pin Liu:** Data curation (equal); formal analysis (equal); investigation (equal). **Chao‐Hung Yeh:** Formal analysis (supporting). **Shu‐Chun Kuo:** Conceptualization (equal); funding acquisition (lead); writing – review and editing (lead). **Ching‐Ping Chang:** Conceptualization (lead); data curation (lead); formal analysis (lead); project administration (lead); writing – original draft (lead); writing – review and editing (lead).

## FUNDING INFORMATION

This study was supported by Chi Mei Medical Center (Taiwan) CMNDMC11101 (T.B.S.), CMFHR11021 (T.B.S.), CMFHR11101 (S.C.K.) and CMFHT 11103 (C.P.C.). The funders had no role in the study design, data collection, analysis, the decision to publish, or the preparation of the manuscript.

## CONFLICT OF INTEREST STATEMENT

The authors declared that they have no competing interests.

## Supporting information


Figure S1.

Figure S2.

Data S1.

## Data Availability

The datasets used and/or analyzed during the current study are available from the corresponding author upon reasonable request.
