# Supplementary figures and images for: Hyperbaric oxygen therapy suppresses hypoxia and reoxygenation injury to retinal pigment epithelial cells through activating peroxisome proliferator activator receptor‐alpha signalling
Source: J Cell Mol Med. 2023 Sep 20;27(20):3189–201. doi: 10.1111/jcmm.17963 (PMC10568664; doi:10.1111/jcmm.17963)

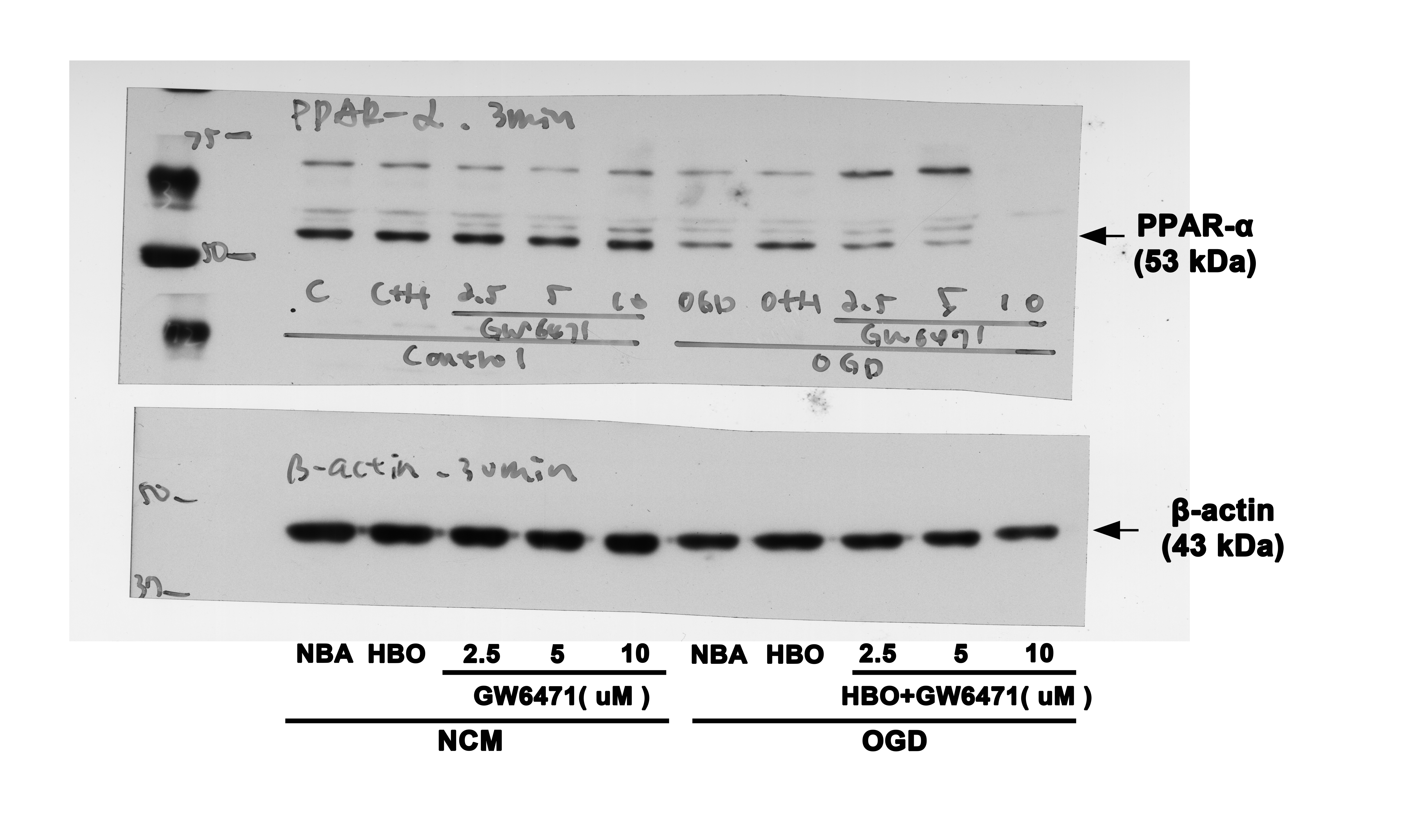

Supplement: Supplementary file 1 — Figure S1. [file JCMM-27-3189-s003.jpg]

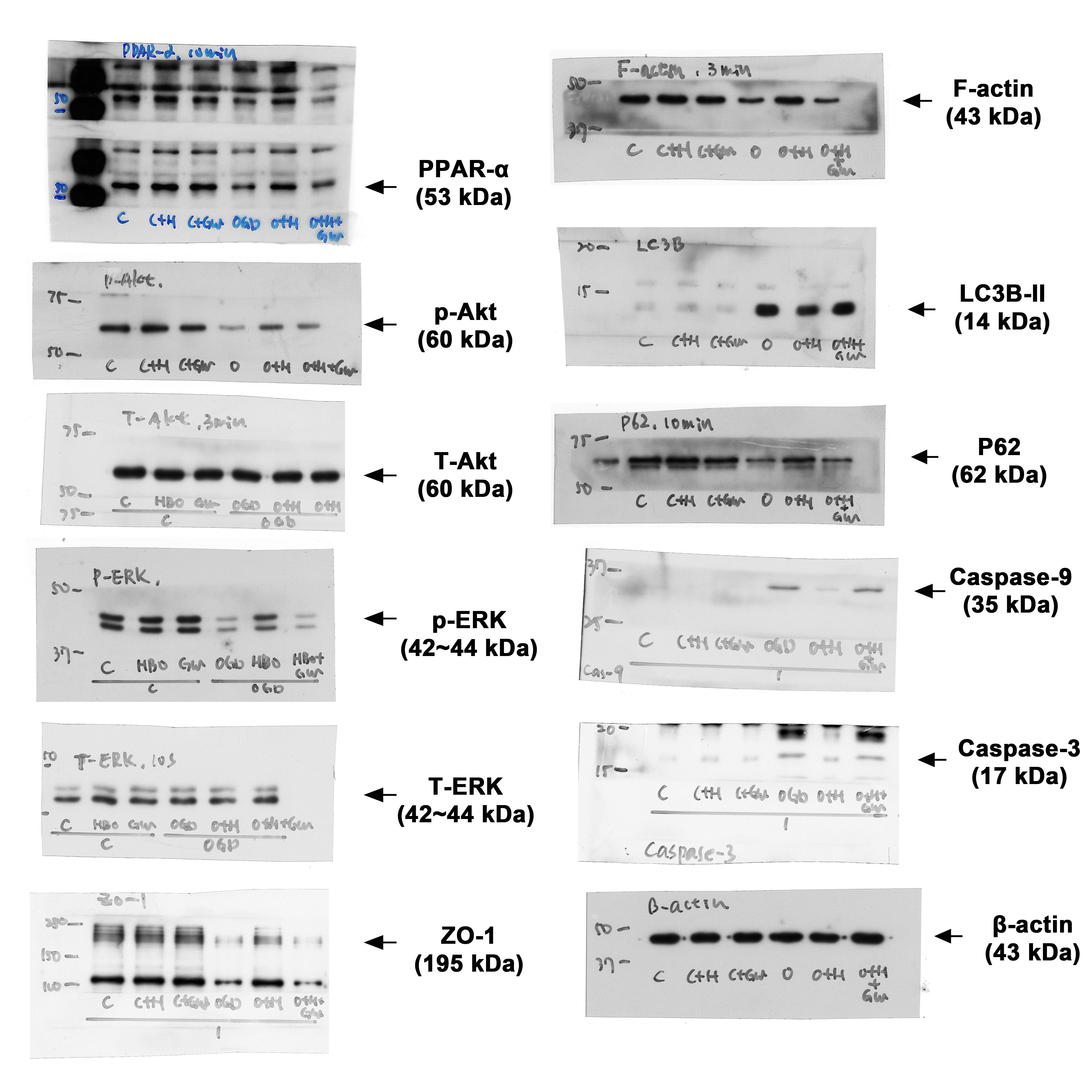

Supplement: Supplementary file 2 — Figure S2. [file JCMM-27-3189-s001.jpg]
