# Supplementary material for: Hyperbaric oxygen therapy suppresses hypoxia and reoxygenation injury to retinal pigment epithelial cells through activating peroxisome proliferator activator receptor‐alpha signalling
Source: J Cell Mol Med. 2023 Sep 20;27(20):3189–201. doi: 10.1111/jcmm.17963 (PMC10568664; doi:10.1111/jcmm.17963)
Supplement: Supplementary file 3 — Data S1. [file JCMM-27-3189-s002.docx]

Supplementary Figure 1: Original western blots for Figure 1 (D). Labels on the right of each x-film scan indicate the antibody used for detection. Dashed red boxes show the regions that were cropped for the figures.

Supplementary Figure 2: Original western blots for Figure 4 (A). Labels on the right of each x-film scan indicate the antibody used for detection. Dashed red boxes show the regions that were cropped for the figures.
